# Shared Decision-Making and Colorectal Cancer Screening Behaviors Among Older Adults With Low Health Literacy

**DOI:** 10.1093/geroni/igab046.1154

**Published:** 2021-12-17

**Authors:** Tamara Cadet, Rebekah Halmo, Siobhan McDonold, Mara Schonberg

**Affiliations:** 1 University of Pennsylvania , Philadelphia, Pennsylvania, United States; 2 Simmons University, Boston, Massachusetts, United States; 3 Division of General Medicine, Beth Israel Deaconess Medical Center, Harvard Medical School, Boston, Massachusetts, United States

## Abstract

National guidelines recommend adults >75 engage in shared decision making (SDM) around colorectal cancer (CRC) screening because of the uncertain benefit to risk ratio. There are no decision tools to support CRC decision making for adults >75 years with low health literacy (LHL). The purpose of this mixed-methods study was to better understand the perspectives of adults >75 with LHL on SDM around CRC screening and to obtain their feedback on an existing higher literacy CRC decision aid. Utilizing the Brief Health Literacy Screening Tool to identify participants with LHL, semi-structured interviews were conducted with 30 adults. Findings indicate that 80% of participants were non-Hispanic Black and 42% had < high school degree. 76% felt they would benefit from CRC screening despite their age. Themes related to CRC screening included lack of knowledge of options and harms, but a desire to understand more to better take care of their health.

